# The combination of metformin and high glucose increased longevity of *Caenorhabditis elegans* a DAF-16/FOXO-independent manner: cancer/diabetic model via *C. elegans*

**DOI:** 10.3389/fendo.2024.1435098

**Published:** 2024-11-04

**Authors:** Şeyda Berk, Ali Cetin, Özgür Ülkü Özdemir, Ayşe Nur Pektaş, Nazan Yurtcu, Sevgi Durna Dastan

**Affiliations:** ^1^ Department of Molecular Biology and Genetics, Faculty of Science, Sivas Cumhuriyet University, Sivas, Türkiye; ^2^ Advanced Technology Research and Application Center (CUTAM), Sivas Cumhuriyet University, Sivas, Türkiye; ^3^ Department of Obstetrics and Gynecology, Haseki Training and Research Hospital Affiliated with the University of Health Sciences, Istanbul, Türkiye; ^4^ Department of Obstetrics and Gynecology, Faculty of Medicine, Sivas Cumhuriyet University, Sivas, Türkiye; ^5^ Department of Biology, Faculty of Science, Sivas Cumhuriyet University, Sivas, Türkiye

**Keywords:** aging, *C. elegans*, glucose, lifespan, lin-35, metformin

## Abstract

**Introduction:**

Sedentary lifestyles and diets with high glycemic indexes are considered to be contributing factors to the development of obesity, type 2 diabetes in humans. Metformin, a biguanide medication commonly used to treat type 2 diabetes, has been observed to be associated with longevity; however, the molecular mechanisms underlying this observation are still unknown.

**Methods:**

The effects of metformin and high glucose, which have important roles in aging-related disease such as diabetes and cancer, were studied in lin-35 worms because they are associated with cancer-associated pRb function in mammals and have a tumour suppressor property.

**Results and Discussion:**

According to our results, the negative effect of high glucose on egg production of lin-35 worms was greater than that of N2 worms. High glucose shortened lifespan and increased body length and width in individuals of both strains. Metformin treatment alone extended the lifespan of N2 and lin-35 worms by reducing fertilization efficiency. However, when metformin was administered in the presence of high glucose, the lifespan of lin-35 worms was clearly longer compared to N2 worms. Additionally, we conclude that glucose and metformin in lin35 worms can extend life expectancy through a DAF-16/FOXO-independent mechanism. Furthermore, the results of this study will provide a new perspective on extending mammalian lifespan through the model organism *C. elegans*.

## 1 Introduction

The impact of longevity-promoting agents, or geroprotectors, on the development of spontaneous tumours may offer crucial insights into the interplay between aging and carcinogenesis (1). Based on current understanding of aging factors, mechanisms, and theories, these pharmacological interventions have occasionally been associated with unfavourable side effects (2, 3). By contrasting the information about the mechanisms of geroprotector action with its effect on tumours, we can better comprehend the interplay between aging and carcinogenesis, two essential biological processes.

Metformin, a biguanide derived from the French lilac plant, *Galega officinalis*, is the most commonly prescribed anti-diabetic medication. It is effective in lowering blood sugar levels with minimal side effects and has received significant attention as a compound with the potential to promote healthy aging (4, 5). Metformin has been shown to have positive effects in the treatment of cardiovascular disease, certain types of cancer, and to enhance cognition in older adults in addition to its anti-diabetic properties (4, 6, 7). The substantial interest in advantages of metformin is heightened by strong evidence that it promotes healthy aging and lengthens life in a range of model organisms (4, 6, 8). Furthermore, compared to matched healthy controls, metformin has been linked to longer survival rates in patients with type 2 diabetes (6, 7).

Although the anti-hyperglycaemic properties of metformin have been well-documented, the complex mechanisms underlying its action remain incompletely understood. Metformin exerts a notable influence on the metabolic machinery, specifically targeting the cellular energy sensor AMPK, mTOR, and the mitochondrial complex I (9). Different molecular processes may be activated by different doses of metformin. The intricate relationship between metformin and physiology is further complicated by its interactions with the microbiome, which can have significant negative effects on health in humans, *Drosophila melanogaster*, and the nematode *C. elegans* (6, 9). The picture that shows up is that metformin interacts with physiology on multiple levels, which could cause a population to respond differently on an individual basis (9).

From *C. elegans* to mammals, the insulin signalling pathway has been highly conserved throughout evolution (10, 11). Mammalian tyrosine kinase receptors bind to insulin and its close homolog IGF-1, blocking the transcription factor FOXO, which is an essential transcriptional regulator of various cellular processes, including metabolism, longevity, stress response, and apoptosis (10–12). Reducing the activity of this pathway through mutation of the *C. elegans* DAF-2 insulin/IGF-1 receptor gene doubles lifespan and slows the aging process (13). Lifespan of numerous organisms have been demonstrated to be regulated by the insulin/IGF-1 signalling (IIS) pathway (10, 11, 14).

Our current understanding of the impact of high glucose and metformin on the *C. elegans* insulin/IGF-1 signalling pathway and lifespan is extremely limited, despite the well-established link between the insulin/IGF-1 signalling pathway and aging in this organism.

By inducing and preserving cellular quiescence, the DREAM (Dp/Retinoblastoma (Rb)-like/E2F/MuvB) transcriptional repressor complex, which is conserved from invertebrates to mammals, acts as the gatekeeper of the mammalian cell cycle (15). The highly conserved DREAM transcriptional repressor complex, which includes Dp, Retinoblastoma (Rb)-like, E2F, and MuvB, mediates this cell cycle quiescence pathway (16). Lin-35, an orthologue of human retinoblastoma protein (pRB), a component of the worm DREAM complex, is a pocket protein found in *C. elegans*, and one of the important mechanisms it plays a role in is apoptosis (17). The finding that lin-35 suppresses the expression of lin-3, a member of the EGF family, raises the possibility that paracrine growth factor signalling suppression is a mechanism by which members of the Rb family slow down cell growth and thereby prevent cancer (18). Thus, it has been proposed that lin-35 function analysis may offer important insights into new Rb family tumours suppression mechanisms (19). Finally, research in non-mammalian systems like *C. elegans* has revealed several additional cell cycle and developmental roles for the E2F-pRb network, some of which have been demonstrated to be connected to pRb functions that have been proposed to be related to cancer in mammals (15). Lin-35 is linked to cancer-related pRb function and has a tumours suppressor effect. Therefore, lin-35 worms were included in this study to investigate the effects of metformin and high glucose, which have important roles in aging-related diseases such as diabetes and cancer.

In current study, we investigated the effects of high glucose, metformin and the combination of the both on *in vivo* fertilisation, lifespan and body size in N2 and lin-35 worms. We also evaluated differential mRNA expression of insulin/IGF signalling (IIS) components associated with key nutrient sensing pathways of the best-validated antiaging interventions. Additionally, the results of this study investigating the combined effects of glucose and metformin on the mutant strain lin-35, which is associated with the tumours suppressor pRb function in mammals, will provide a new perspective on extending mammalian lifespan through the model organism *C. elegans*.

## 2 Materials and methods

### 2.1 *C. elegans* and *Escherichia coli* strains

Wild-type (N2 Bristol) and mutant AWR58-lin-35 (kea7[lin-35p::degron::GFP::lin-35]) I; keaSi10 II *C. elegans* strains and OP50 strain of E. coli as food source of *C. elegans* were purchased from the Caenorhabditis Genetics Centre (CGC), University of Minnesota (Minneapolis–St. Paul, MN 55455, U.S.A).

### 2.2 *E. coli* OP50 growth conditions

*E. coli* OP50 strain is used as a food source for *C. elegans* and is grown monoxenically. *E. coli* OP50 is a uracil auxotroph whose growth is limited to NGM. A limited bacterial culture is preferred because it allows easier observation and better mating of worms (20). Starting culture is used to isolate single colonies on a streaked plate of a rich medium such as Luria-Bertani (LB) agar [10 g Bacto-tryptone, 5 g Bacto-yeast, 5 g NaCL, 15 g agar, prepared with H_2_O for 1 liter, pH 7.5]. Aseptically inoculate a single colony from the plate into a rich solution of LB Broth [10 g Bacto-tryptone, 5 g Bacto-yeast, 5 g NaCl, 1 liter with H_2_O, pH adjusted to 7 using 0.1 M NaOH]. It can be stored for several months at room temperature. Incubate the cultures overnight at 37°C in a shaking incubator. After incubation, check for growth, which is characterised by a hazy haze in the medium. The *E. coli* OP50 solution is then ready to be used for seeding NGM petri plates. The *E. coli* OP50 petri plate and the liquid culture should be stored at 4°C and can remain usable for several months (21).

### 2.3 C. elegans culture

Worms were maintained according to Brenner’s instructions previously described (20). Obtaining axenised *C. elegans* eggs and synchronous cultures of *C. elegans* were performed according to the method previously described (22). In our study, the lin-35 strain purchased from CGC was treated with auxin before drug application, as it was stated that it needed to be auxinised, especially for the degradation of DREAM proteins. After synchronisation of lin-35 worms, worms were placed in 1×M9 buffer with 1mM auxin and *E. coli* was added. Worms were placed in the moist and hot box prepared beforehand and left for 30 minutes (23). After incubation, drug applications of auxinised lin-35 worms were carried out.

### 2.4 Drugs and reagents

The metformin was purchased from Biosynth, Turkey (1115-70-4) dissolved in ddH_2_O as concentrated 1.2 M stock solution (stored at −20 °C). Auxin was purchased from Bio Basic, Turkey (Q7202130) dissolved in 1X M9 buffer as concentrated 1 M stock solution stored at +4 °C.

### 2.5 Fertilisation assay

For the assay, the age-matched adult worm (three worm per well for each strains) were transferred to 24-well plates containing 1 mL/well S Medium [1L S Basal, 10 mL 1 M potassium citrate pH: 6, 10 mL trace metals solution (1.86 g disodium EDTA, 0.69 g FeSO_4_ •7 H_2_O, 0.2 g MnCl_2_•4 H_2_O, 0.29 g ZnSO_4_•7H_2_O, 0.025 g CuSO_4_•5 H_2_O, dissolved in 1L H_2_O), 3 mL 1 M CaCl_2_, 3 mL 1 M MgSO_4_] with 3µL of *E. coli* OP50 and increasing concentrations of metformin (1mM, 10mM, 25mM, 50mM and 100mM) in presence of 25mM glucose and cultured at 20°C. The number of eggs laid in each well for 10 days was counted under microscope. The experiments were repeated twice, and each experiment was performed in quadruplicate (24).

### 2.6 Lifespan assays

The L4 stage worms (~12 worms per well for each strains) were transferred to 24-well plates containing 1 mL/well S Medium with 3µL of *E. coli* OP50, 25mM glucose and increasing concentrations of metformin (1mM, 10mM, 25mM, 50mM and 100mM), and worms were cultured at 20°C for lifespan assay. FUDR (5-Fluoro-2’-deoxyuridine) was used to prevent reproduction. The medium was changed every 7 days and worms counted under the microscope every other day. Animals that did not move after being gently nudged to move the plate were deemed dead. For body size analyses of worms, 0-, 7-, and 14-day photomicrographs were obtained using the Zeiss Axiovert A1 with Zeiss Digital Microscope Camera (AxioCam ICc 5). Body length was determined using ImageJ software. Data were analysed using GraphPad Prism. The experiments were repeated twice, and each experiment was performed in duplicate (22).

### 2.7 RNA isolation and cDNA synthesis

Following synchronisation, about 3000 L4 stage worms from two distinct generations of N2 and lin-35 were placed in 24-well plates with 1 mL/well S Medium and 3µL of *E. coli* OP50. The worms were then cultivated for seven days at 20°C. Following a 7-day incubation period with either glucose or metformin, worm samples were prepared for RNA isolation using the previously described procedure (25). Total RNA was obtained using a commercially available kit, following the manufacturer’s instructions (HibriGen Biotechnology, Istanbul, Turkey, Total RNA Isolation kit; Cat. No: MG-RNA-01-100). The iScript cDNA Synthesis Kit (Bio-Rad, Hercules, CA, USA; Cat. No: 1708891) was used to synthesise cDNA with 500 ng/µL of RNA, following the manufacturer’s guidelines. The generated cDNA was either analysed immediately by qPCR or stored at +4°C for future use.

### 2.8 Quantitative real-time PCR

Expression in N2 and lin-35 at L4 stage worm of components of the IIS system (*daf-16a, daf-16b, daf-16d/f, total daf-16* and *daf2*) mRNA was evaluated by RT-qPCR. Following the manufacturer’s instructions for real-time amplifications, quantitative PCR was carried out using the StepOnePlus™ Real Time PCR equipment (Applied Biosystems, USA) and the SYBR green assay. In a total reaction volume of 20 µL, the experiment was carried out using 10 µL of BlasTaqTM 2X qPCR Master Mix (Abm, Canada, USA), 0.5 µL of each primer, and 2 µL of cDNA template. Primer details are given in **Table 1**. Every reaction was conducted using 96-well plates. The real-time instrument was used to conduct PCR in accordance with the previously outlined procedure (22). In the study our research group conducted, the most stable genes for N2 and lin-35 strains were found to be *act-1* and *cdc-42* reference genes (29). Therefore, the previously reported approach was employed to normalise mRNA levels using *act-1* and *cdc-42* (30). The comparative threshold approach, 2^-ΔCt^, was utilised to compute the relative expression of genes. Primers were purchased from BMLabosis (Ankara, Turkey). Ct values greater than 35 produced inconsistent findings and were considered below the assay’s detection limit.

**Table 1:** Primer sequences for RT-qPCR.

Gene symbol	Primer sequence	Reference
*daf-16a*	F: 5’-CACCGGATGATGTGATGATG-3’ R:5’-CTCCCGTATAGGTCAGCATC-3’	(26)
*daf-16b*	F: 5’-CCTATTCGGATATCATTGCC-3’ R: 5’-GGATCGAGTTCTTCCATCCG-3’	(26)
*daf-16d/f*	F: 5’-CAATCTCGACCTCCATCAAC-3’ R:5’-CCCGTATAGGCTAGTTCTTC-3’	(26)
*total daf-16*	F: 5’-AAGCCGATTAAGACGGAACC-3’ R:5’-GTAGTGGCATTGGCTTGAAG-3’	(26)
*daf-2*	F:5’-GTACAGCCGTGTGCCTCAATAGTC-3’ R:5’-ATTGTCAGCGAACCTTCCACCAC-3’	(27)
*act-1*	F: 5’-CTCTTGCCCCATCAACCATG-3’ R: 5’-CTTGCTTGGAGATCCACATC-3’	(28)
*cdc-42*	F: 5’-CTGCTGGACAGGAAGATTACG-3’ R: 5’-CTCGGACATTCTCGAATGAAG-3’	(28)

### 2.9 Statistical analysis

All experiments were repeated at least twice. The outcomes of the repeated experiments were comparable. The statistical software GraphPad Prisim 6.0 (GraphPad software, San Diego, CA) was utilised for the analysis. For survival, log-rank tests were used. A one-way ANOVA test was used to compare statistical results between groups. When significant differences were discovered, the Newman-Keuls test was used to compare groups. p<0.05 was considered statistically significant in all analyses. The data is shown as mean SEM.

## 3 Results

### 3.1 Effects of glucose and metformin on *C. elegans* fertilisation

**Figure 1:**
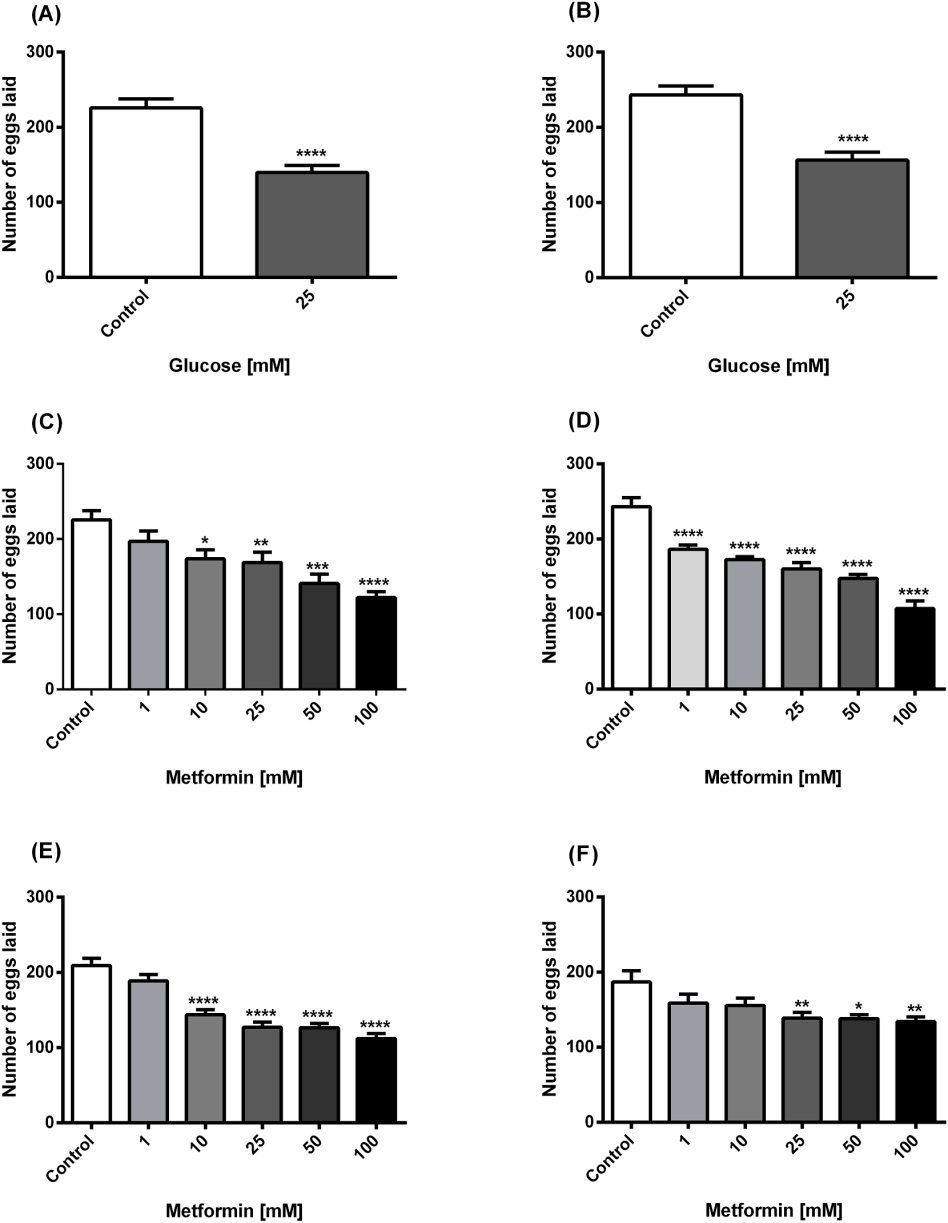
Panels **(A, B)** The effect of high glucose on egg-laying in N2 **(A)** and lin-35 **(B)** worms. Panels **(C, D)** Effect of metformin on egg-laying in N2 **(C)** and lin-35 **(D)** worms in the absence of glucose. Panels **(E, F)** Effect of metformin on egg-laying in N2 **(E)** and lin-35 **(F)** worms in the presence of 25mM glucose. The experiments were carried out on synchronised populations. Worms were assessed to be exposed to only glucose or to metformin in the presence/absence of glucose. During the egg stage, synchronised populations of wild-type and mutant *C*. *elegans* were transplanted to 24-well plates containing liquid medium with glucose (25mM) or increasing concentrations of metformin (1-100 mM) with or without glucose. Significance relates to a comparison of egg-laying behaviour in *C*. *elegans* exposed to glucose or metformin with or without glucose versus control. Control groups in panels **(A, B)** contain no glucose, panels **(C, D)** contain no metformin, while controls in panels **(E, F)** contain only 25 mM glucose. The number of eggs laid by each worm was counted every day. Values represent the mean ± SEM of two independent experiments in quadruplicate. *p < 0.05, **p<0.001, ***p<0.001, ****p<0.0001; n=3.

According to the results of the effect of glucose on the egg-laying capacity of worms, it was observed that 25mM glucose significantly reduced the number of eggs laid by both N2 and lin-35 worms compared to the control (**Figures 1A, B**). According to the results of the effects of metformin on egg laying of worms, a significant inhibitory effect of metformin was observed at the four highest concentrations (10, 25, 50 and 100 mM) in N2 worms treated only with metformin (**Figure 1C**), while it had a significant inhibitory effect at the all concentrations (1, 10, 25, 50 and 100 mM) in lin-35 worms (**Figure 1D**). In both strains, the strongest inhibitory effect was observed in the presence of 100mM metformin. On the other hand, the egg-laying capacity of N2 worms treated with both 25mM glucose and different concentrations of metformin decreased significantly in the last four concentrations (10, 25, 50 and 100 mM) (**Figure 1E**). The inhibitory effect at these four concentrations was statistically stronger compared to treatment with metformin alone in N2 worms. On the other hand, when metformin was applied at increasing concentrations in the presence of 25mM glucose in lin-35 worms, the inhibitory effect remained weaker than the condition in which only metformin was applied. The inhibitory effect of metformin with high glucose on egg laying of lin-35 worms was significant at concentrations of 25, 50, and 100 mM (**Figure 1F**).

### 3.2 Screening of glucose and metformin for lifespan effects of *C. elegans*

**Figure 2:**
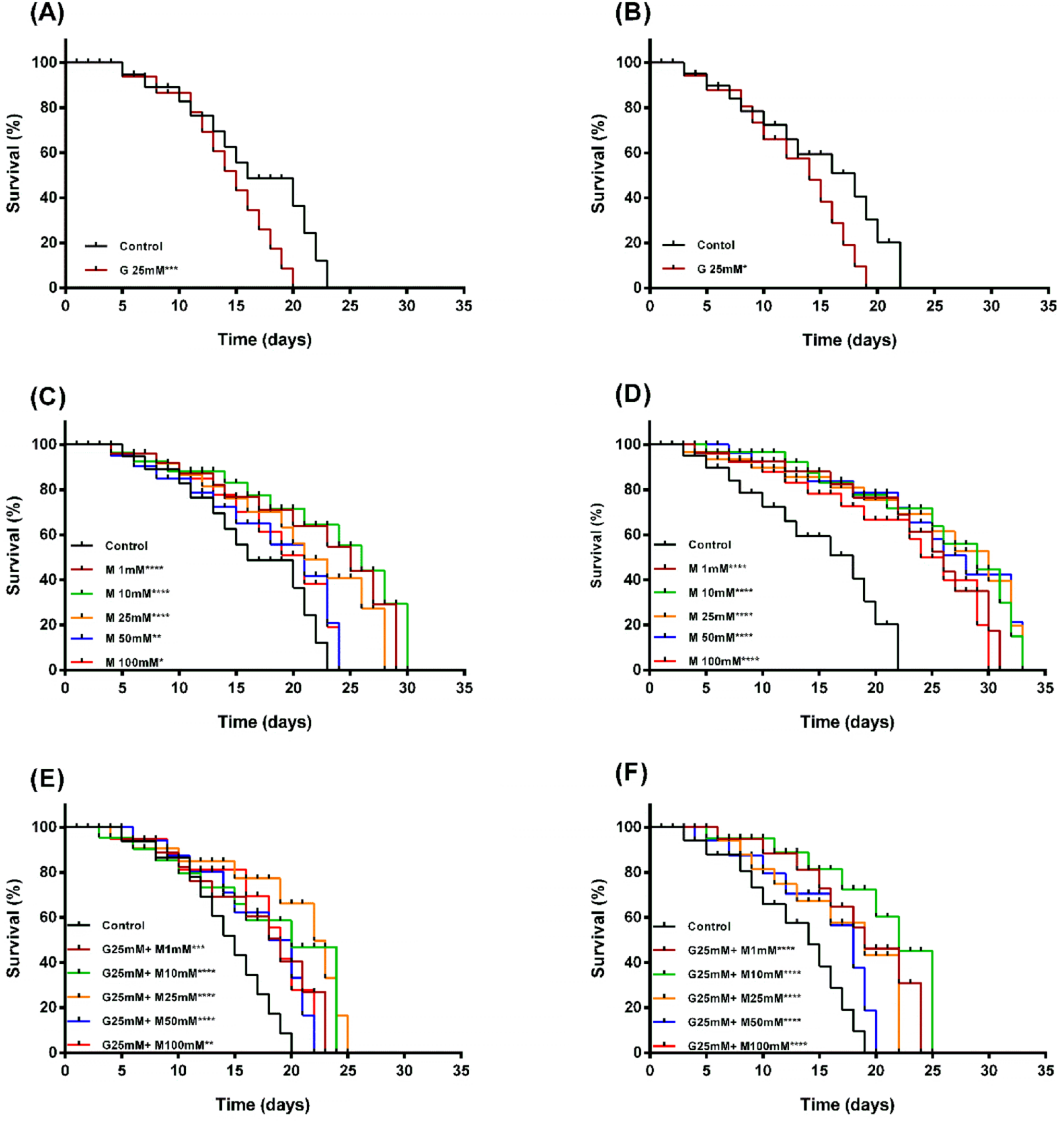
Panels **(A, B)** Lifespan survival curves of N2 **(A)** and lin-35 **(B)** worms in the presence of glucose. Panels **(C, D)** Lifespan survival curves of N2 **(C)** and lin-35 **(D)** worms in the presence of metformin without glucose. Panels **(E, F)** Lifespan survival curves of N2 **(E)** and lin-35 **(F)** worms in the presence of metformin and 25mM glucose. The experiments were carried out on synchronised populations. The synchronised populations of wild-type and mutant *C*. *elegans* were transplanted to 24-well plates containing liquid medium with glucose (25mM) or increasing concentrations of metformin (1-100mM) with or without glucose. Significance relates to a comparison of lifespan in *C. elegans* exposed to glucose or to metformin with or without glucose versus control. Control groups in panels **(A, B)** contain no glucose, panels **(C, D)** contain no metformin, while controls in panels **(E, F)** contain only 25 mM glucose. Labels state the name and concentration of the drug (G, Glucose; M, Metformin). Values represent the mean ± SEM of two independent experiments in quadruplicate. *p < 0.05, **p<0.001, ***p<0.001, ****p<0.0001.

**Figure 3:**
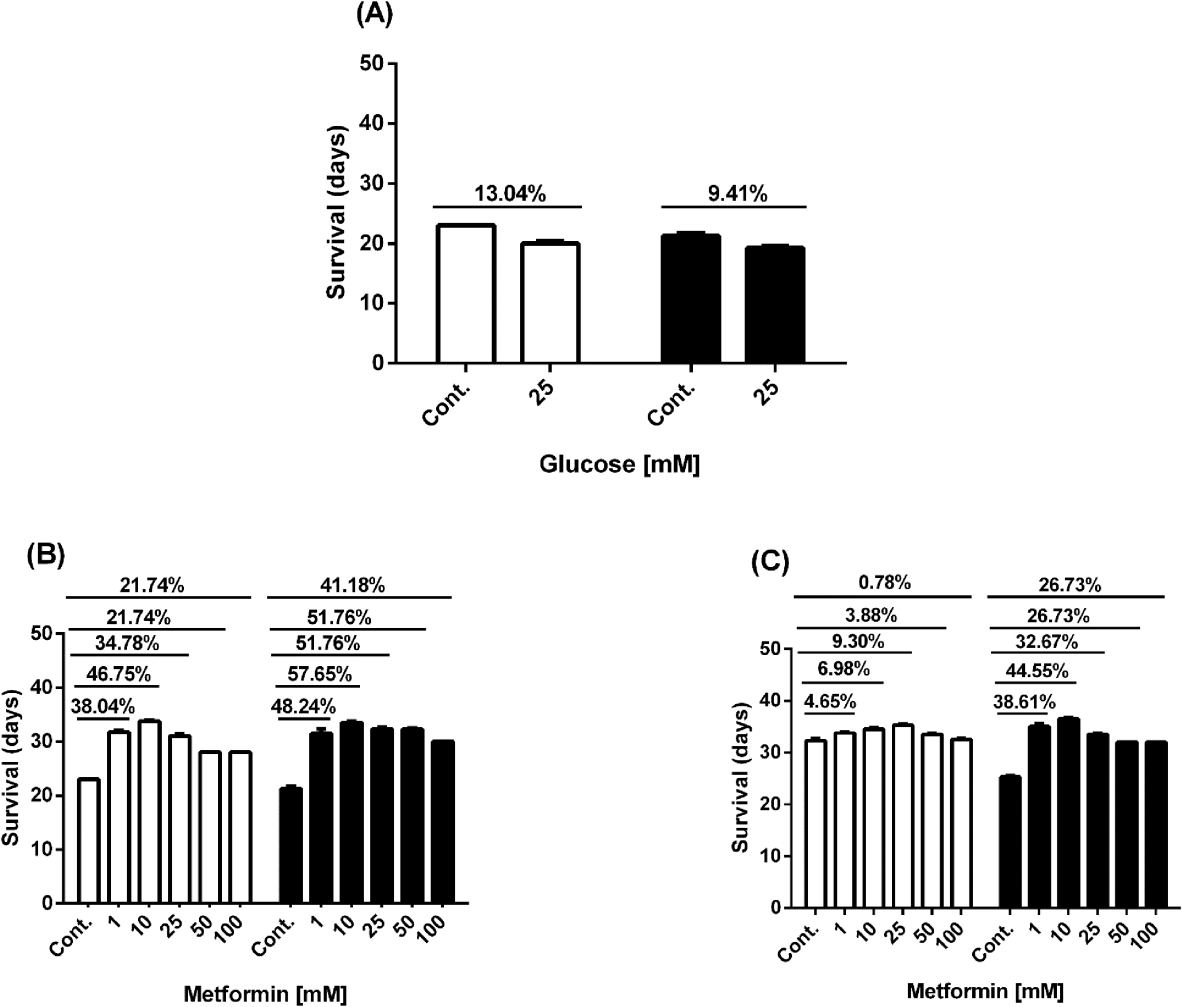
Graph showing the % increase in lifespan of N2 (white bars) and lin-35 (black bars) worms in the presence of glucose **(A)**, metformin without glucose **(B)** and metformin with 25mM glucose **(C)** compared to control.

We confirmed the effects of high glucose and metformin in the presence or absence of high glucose on worm lifespan. Our findings showed that the lifespan of N2 and Lin-35 worms decreased under high glucose condition, but it was extended when treated with metformin alone or metformin combined with high glucose (**Figures 2**, **3**). 25mM glucose reduced the average lifespan of N2 and lin-35 worms by 13.04% and 9.41%, respectively (**Figuress 2A, B**, **3A**). Metformin at concentrations of 1, 10, 25, 50, and 100 mM increased the average lifespan of N2 worms by 38.04%, 46.75%, 34.78%, 21.74%, and 21.74%, respectively (**Figures 2C**, **3B**, white bars), while lin-35 worms increased by 48.24%, 57.65%, 51.76%, 51.76%, and 41.18%, respectively (**Figures 2D**, **3B**, black bars). In the presence of 25mM glucose, 1, 10, 25, 50, and 10 mM metformin increased the lifespan of N2 worms by 4.65%, 6.98%, 9.30%, 3.88%, and 0.78%, respectively (**Figures 2E**, **3C**, white bars), the lifespan of lin-35 worms was increased by 38.61%, 44.55%, 32.67%, 26.73%, and 26.73%, respectively (**Figures 2F**, **3C**, black bars). When glucose alone was applied, the lifespan of N2 worms was reduced at a higher rate than that of lin-35 worms. On the other hand, when metformin alone was administered at all concentrations, the lifespan of lin-35 worms increased more than that of lin-35 worms. When both 25 mM glucose and different concentrations of metformin were administered together, the lifespan of lin-35 worms increased more than that of N2 worms at all concentrations. As expected, the extension of the lifespan of the worms was observed to be independent of concentration. That is, as the inhibitor concentration increased, a parallel increase in life span was not observed (**Figures 2**, **3**).

### 3.3 Body size measurement

**Figure 4:**
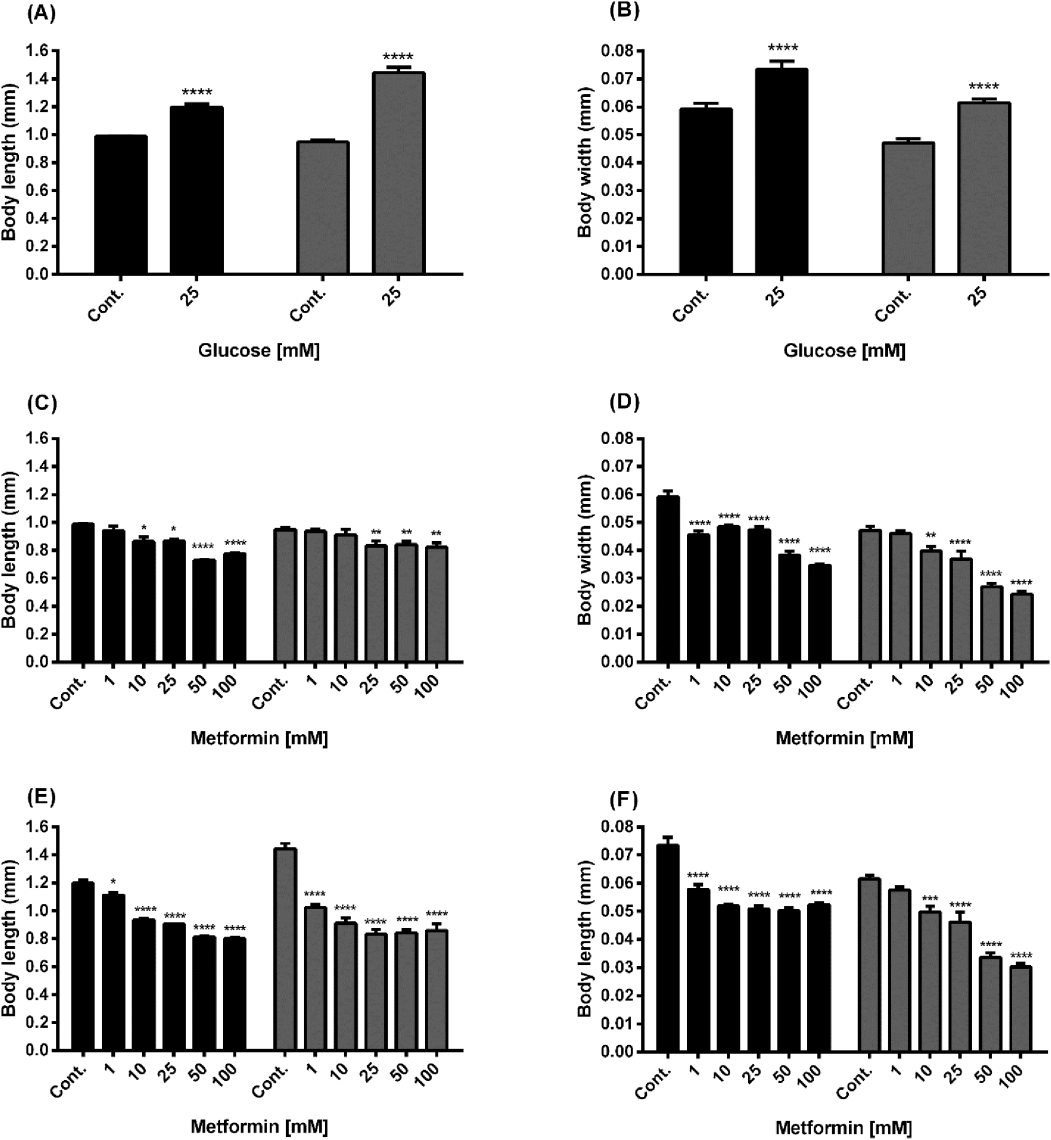
Body size of wild-type N2 (black bars) and mutant lin-35 strains (grey bars) grown in S-medium including only glucose or different concentrations of metformin in presence or absence of glucose. Panels **(A, B)** Body length **(A)** and width **(B)** of N2 and lin-35 worms grown in S-medium including high glucose compared with control. Panels **(C, D)** Body length **(C)** and width **(D)** of N2 and lin-35 worms grown in S-medium including metformin without glucose. Panels **(E, F)** Body length **(E)** and width **(F)** of N2 and lin-35 worms grown in S-medium including metformin and 25mM glucose. Body length and width size derived N2 and lin-35 worms after 14 days is in mm using ImageJ software. Values represent the mean ± SEM of two independent experiments in duplicate. *p < 0.05, **p<0.001, ***p<0.001, ****p<0.0001.

To investigate the effect of metformin and high glucose on the body size of N2 and lin-35 worms, we measured their body length and width after 14 days (**Figure 4**). A significant increase in both body length and body width was observed in N2 and lin-35 worms exposed to 25 mM glucose (**Figures 4A, B**). The body length of N2 worms exposed to metformin alone significantly decreased at 10, 25, 50 and 100 mM metformin concentrations (**Figures 4C, D**, black bars), whereas the length of lin-35 worms significantly decreased in the presence of 25, 50, and 100 mM metformin (**Figure 4C**, grey bars). The body width of N2 worms treated with only metformin decreased significantly in the presence of 1, 10, 25, 50 and 100 mM metformin (**Figure 4D**, grey bars), while in lin-35 worms at 10, 25, 50 and 100 mM metformin. On the other hand, when metformin was administered in the presence of 25 mM glucose, the body size (length and width) of both N2 and lin-35 worms decreased significantly at all metformin concentrations (except body width of lin35 at 1mM metformin) (**Figures 4E, F**).

### 3.4 mRNA expression of components of the IIS system in *C. elegans*

**Figure 5:**
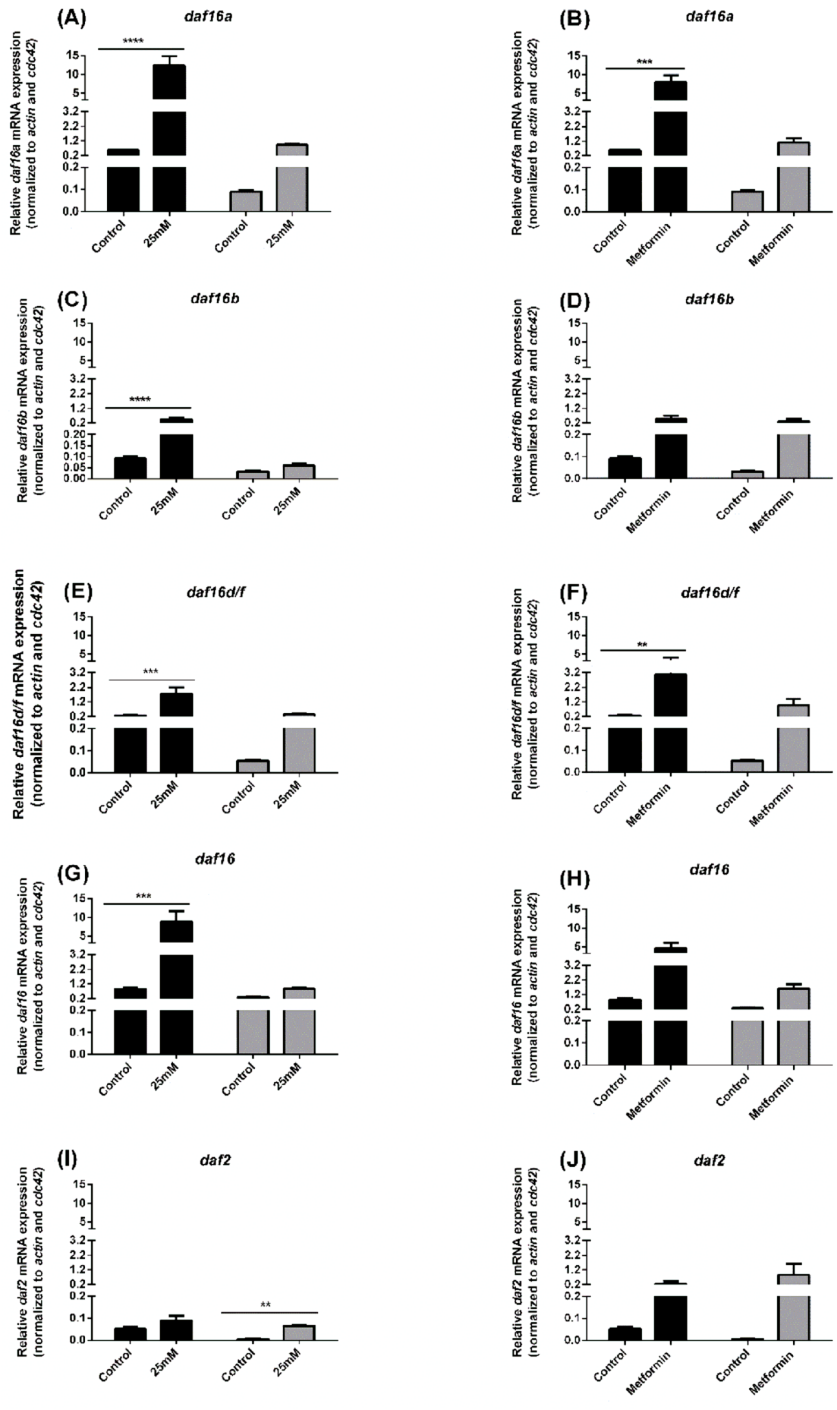
*daf-16a, daf-16b, daf-16d/f, total daf-16* and *daf2* mRNA expression in N2 (black bars) and lin-35 (grey bars) worms in presence of 25mM glucose **(A, C, E, G, I)** or presence of 100 mM metformin **(B, D, F, H, J)**. *daf-16a, daf-16b, daf-16d/f, total daf-16* and *daf2* mRNA expression was measured relative to the levels of the house-keeping genes *cdc-42* and *act-1*. Values represent the mean ± SEM of two measurements from two independent experiments. **p<0.01, ***p<0.001, ****p<0.0001.

In N2 (**Figure 5**, black bars) and lin-35 (**Figure 5**, grey bars) cultured in S-medium containing 25mM glucose (**Figures 5A–E**) or 100 mM of metformin, mRNA expression of the *daf-16a, daf-16b, daf-16d/f, total daf-16* and *daf-2* were measured by quantitative RT-PCR.

Compared with control, *daf-16a, daf-16b, daf-16d/f, total daf-16* mRNA expression was significantly increased in N2 worms cultured with 25 mM glucose, whereas there was no significant increase in lin-35 worms (**Figures 5A–D**). On the other hand, while there was no significant difference in *daf-2* mRNA expression in N2 worms compared to the control, an increase was observed in lin-35 worms (**Figure 5E**). While *daf-16a, daf-16d/f* and *daf-2* mRNA expression was significantly increased in N2 worms cultured with 100 mM metformin compared to control, a significant increase in mRNA expression of all IIS components (*daf-16a, daf-16b, daf-16d/f, total daf-16* and *daf2*) was observed in lin-35 worms (**Figures 5F–J**).

## 4 Discussion

Metformin is a lipophilic biguanide that promotes peripheral glucose utilisation while suppressing hepatic gluconeogenesis. Due to its safety, effectiveness and tolerability, it is a first-line medication for Type 2 diabetic patients who need to control their blood sugar levels (31). A potential anti-tumorigenic effect of metformin is exerted by activating AMP-kinase, which mediates inhibition of the mammalian target of rapamycin (mTOR) (32). Results from several clinical studies have shown that type 2 diabetes patients treated with metformin may have a lower risk of cancer (31). Therefore, in this study, we tried to elucidate the relationship between metformin, diabetes, and cancer on the model organism *C. elegans*. The best validated antiaging studies have been demonstrated in the insulin/IGF signalling (IIS) and rapamycin target (TOR) pathways, two major nutrient sensing pathways (33). The insulin/insulin-like growth factor (IGF) signalling pathway to mTOR is essential for the survival and growth of normal cells and also contributes to the formation and progression of cancer (34). The lin-35 strain present in the current study is the orthologue of pRb in mammals and has a tumours suppressor effect by inhibiting mTOR signalling (18). In this study, the effect of high glucose and metformin in the presence and absence of high glucose on the lifespan of lin-35 worms with Rb mutation was investigated and whether this effect was through the insulin signalling pathway. This effect was also investigated in wild-type N2 worms to reveal the difference in effect with lin-35 worms. Thus, this model created in the *C. elegans* model organism will provide a new perspective to elucidate the relationship between tumours, diabetes, and aging. To our knowledge, there have been no previous studies on how the combination of metformin and high glucose affects fertilisation and survival for *C. elegans*. Therefore, the results of this study investigating the combined effects of glucose and metformin on the mutant strain lin-35, which is associated with the tumours suppressor pRb function in mammals, will provide a new perspective on mammalian life extension through the model organism *C elegans*. For this purpose, we examined the effects of high glucose, metformin, and combination of both on fertilisation. Self-reproducing hermaphrodites of *C. elegans* store sperm in spematheca and produce oocytes. Hermaphrodites store fertilised eggs in their uteruses during the first day of the L4/adult molt, so a young adult hermaphrodite will have 10-15 eggs stored in their uterus at any given time (Schafer, 2005). We found that the number of days to fertility of N2 and lin-35 worms treated with high glucose was shortened (1 day) compared to the control group. On the other hand, we found that when only metformin was administered, it prolonged the fertilisation period of N2 and lin-35 worms, resulting in egg-laying for approximately 3 and 2 more days, respectively. In N2 and lin-35 worms treated with metformin and high glucose, this period was extended by 2 and 1 days, respectively. In both conditions (metformin alone or metformin in the presence of high glucose), the number of days to egg-laying was longer in N2 worms than in lin-35 worms. In addition, when only metformin was applied, the decrease in the number of eggs of lin-35 worms was stronger, whereas when high glucose and metformin were applied, a higher inhibitory effect was observed in N2 worms. Furthermore, under conditions where high glucose, metformin, or both are administered, the relative number of progeny produced per day in N2 and lin-35 worms is reduced. Therefore, we hypothesised that high glucose, metformin, and both affect the reproduction of worms and that this is probably due to defects in egg-laying capacity. Our results on the effect of only high glucose treatment on fertility in N2 worms are consistent with those reported by Teshiba et al., Lu and Qiu, Alcántar-Fernández et al. (35–37) and our results regarding metformin treatment were consistent with the results of Onken and Driscoll, Cabreiro et al. (38, 39). On the other hand, in this study, the combined effect of high glucose and metformin on the fertilisation of N2 worms was revealed for the first time. Additionally, our results regarding the effects of high glucose, metformin, and both on fertility of lin-35 worms are reported for the first time with this study. In these studies investigating the effect of glucose on *C. elegans* fertilisation (35–37), it was generally studied on NGM agar at very high concentrations (100 mM), which create toxic effects for the organisms. This is due to the high bioavailability of administered glucose by the worms. Since the high effectiveness of bioavailability in drug applications on worms cultured in NGM agar has always been controversial, it has been recommended to apply drugs at high concentrations for high efficiency of bioavailability (40). However, high drug applications at concentrations well above these normal values, cell culture and patient studies fail to show parallelism. Therefore, in our current study, worms were exposed to glucose and metformin in liquid media in order not to create any doubt about bioavailability, since glucose and metformin spread more homogeneously in liquid media and the bioavailability of worms is high in liquid media. It has also been reported that metformin is unstable at high temperatures (41). The drug must be added before the NGM agar cools and gels, so this appears to be a disadvantage in applications of temperature-sensitive drugs such as metformin. In such cases, drug application in *C. elegans* liquid culture prevents such disadvantages.

In previously reported studies, *C. elegans* lifespan was also shortened in the presence of high glucose (42, 43). In these two studies, the effects of adding 2% glucose (equivalent to approximately 11M glucose) to NGM agar on the lifespan of worms were investigated and they found that the lifespan of worms treated with 2% glucose was significantly shortened. In the study conducted by Schlotterer and colleagues, 40mM glucose was applied to create an environment similar to hyperglycaemic conditions in diabetic patients, since a glucose treatment of 40 mmol/L in agar resulted in a glucose concentration of 14 mmol/L in the whole body extract of *C. elegans* and they observed that the lifespan of worms decreased (44). According to our longevity analysis results, both wild-type N2 and mutant lin-35 worms shortened their lifespan in the presence of 25 mM glucose. We also found that the lifespan of N2 worms was further extended after 25 mM glucose treatment compared to lin-35 worms (**Figure 1**). As a result, consistent with the results of previous studies investigating the effect of glucose on longevity (even though very high glucose concentrations were studied), in our current study, high glucose reduced both the egg-laying efficiency and lifespan of N2 and lin-35 worms.

Experiments are still needed to clarify the molecular processes via which metformin confers health advantages, despite the considerable potential of metformin to support healthy aging (45–47). In order to close this knowledge gap, we first investigated the possibility that metformin could have physiological effects in *C. elegans* that are comparable to those seen in mammals. A review of additional studies carried out with the same objective was conducted. In one of these studies, conducted by Onken and Driscoll, the mean survival of wild type animals grown with 50 mM metformin has been reported to increase by nearly 40%, while wild type cultures grown with 1 mM and 10 mM metformin showed a survival rate comparable to controls grown without metformin (38). In addition, it was demonstrated by Cabreiro and associates that metformin at 25, 50, and 100 mM prolonged the average lifespan of N2 worms by 18%, 36%, and 3%, respectively. Additionally, they discovered that the ability metformin to prolong lifespan was dependent on living *Escherichia coli*, and that without these microorganisms, metformin reduced lifespan of *C. elegans* (39). Another study conducted by Onken and colleagues showed that exposure to 50 and 70 mM metformin extended the lifespan of N2 worms by 35% and 41%, respectively (9). In current study, we monitored survival rates of *C. elegans* occurring at increasing concentrations of metformin. Based on the results of the effectiveness of metformin alone on longevity, metformin in the presence of all concentrations extended the lifespan of both wild-type N2 and mutant lin-35 worms. We also found that the lifespan of lin-35 worms was further extended after metformin treatment compared to N2 worms (**Figures 2**, **3**). As a result, only metformin treatment extended the lifespan of N2 and lin-35 worms by reducing fertilisation efficiency. Additionally, lifespan of N2 worms increased more at lower concentrations (1 and 10mM), whereas lifespan of lin-35 worms were observed at higher metformin concentrations (10, 25 and 50mM) than N2 worms. We hypothesised that metformin administration at higher concentrations may have had a toxic effect on N2 worms. However, when metformin was administered in the presence of high glucose, the lifespan of lin-35 worms was clearly longer compared to N2 worms. Under conditions where high glucose and metformin were administered together, the decrease in the rate of increase in lifespan in N2 worms administered only metformin was sharper than in lin-35 worms. This seems to be consistent with the data that metformin use in also patients with diabetes or aging-related diseases slows down aging and extends lifespan in *C. elegans* (48), we aimed to determine whether growth on high-glucose diets causes a change in body length and width. Alcántar-Fernández and colleagues observed that worms reared at 20, 40, 80, or 100 mM glucose were longer and thicker than control worms (37). Consistent with these results, in our current study, there was a significant increase in the length and width of both N2 and lin-35 worms when worms were treated with high glucose.

In accordance with studies that analysed the body size of worms exposed to metformin and showed that metformin caused a decrease in body size (49), in our study, metformin caused significant decreases in the body size of N2 and lin-35 worms. In present study, only after metformin treatment, significant decreases were observed in both body length and width of wild-type N2 and lin-35 worms, especially those exposed to 50 and 100 mM metformin concentrations, while when metformin was applied in the presence of high glucose, it was possible to observe a decrease in the body size of the worms at all metformin concentrations. In addition, when 50 and 100 mM metformin was administered alone and in combination with glucose, the greatest decrease in body width was observed, especially in lin-35 worms.

Furthermore, a study with mice showed that long-term treatment with metformin (0.1% w/w in diet) extends health span and lifespan in mice, while a higher dose (1% w/w) was toxic. Additionally, metformin has been reported to cause weight reduction in mice (50). In another study conducted by Anisimov et al., after metformin was administered to male and female mice, a slight increase in mean and maximum lifespan was observed in females, while a decrease in mean and maximum lifespan was observed in male (51). Another study reported that body weight decreased significantly in the group of mice with metformin in their diet compared to the control group (52). Similar to the results of previous studies in *C. elegans* and mice, in our current study, high glucose increased the body size of worms and shortened their lifespan; metformin treatment extended the lifespan of the worms and reduced their body size and fertility.

In *C. elegans*, daf-16 activation inhibits both aging and tumour growth. These two processes are essentially linked since tumour rates grow as the animal ages (1). It has been discovered that Daf-16/FOXO-regulated genes that affect the insulin/IGF-1 pathway as influencing the formation of *C. elegans* cancer formation (53). Although some of these genes are required for the full effect of daf-2 mutations on cell death, none appears to be required for the full effect on cell division. Since both increased apoptosis and decreased cell proliferation contribute to the tumour-protective effects of daf-2 mutations, downstream genes therein act cumulatively, as do the downstream targets of daf-16 is likely to act in a cumulative manner to influence tumour growth (54). Almost half of the genes that affect tumour growth have also been shown to affect the survival of animals without tumours. This suggests that the ability of the insulin/IGF-1 pathway to couple longevity and tumour resistance lies downstream of DAF-16 (55).

Downregulation of the insulin signalling system enhances *C. elegans* longevity and locomotor healthspan in a FOXO/DAF-16-dependent manner (13, 56–58). Insulin/IGF-1 signalling suppresses the transcriptional activity of DAF-16/FOXO (12). This inhibition happens as follows: Increased daf-2 signalling leads to the phosphorylation of age-1, pdk-1, and akt-1/2, which keeps the daf-16 protein in the cytosol and deactivates DAF-16/FOXOs. In the absence of daf-2, age-1, and akt-1/2, as in food shortage, daf-16 migrates to the nucleus and stimulates the expression of genes relevant to lifespan (59). When insulin/IGF-1 signalling is decreased, lifespan doubles, and this needs daf-16 (57). Deleting daf-16 in otherwise healthy animals accelerates tissue aging and reduces lifespan by approximately 20% (57, 60). We hypothesised that glucose could reduce the lifespan of *C. elegans* by affecting elements of the insulin/IGF-1 signalling system, since glucose enhances insulin secretion in mammals. According to our mRNA expression analysis results, in the presence of 25 mM glucose, *daf-16a, daf-16b, daf-16d/f*, and *total daf-16* mRNA expression increased significantly in N2 worms compared to the control group, while no statistically significant change in *daf-2* mRNA expression was observed. In lin-35 worms, while there was no significant change in *daf-16a, daf-16b, daf-16d/f* and *total daf-16* mRNA expression, a statistically significant increase was observed in *daf-2* mRNA expression. These results support the concept that glucose treatment for N2 worms upregulates the insulin/IGF-1 signalling pathway (42), which counteracts the lower activity of the insulin/IGF-1 receptor in these animals, but do not appear to be consistent for lin-35 worms.

Furthermore, to determine if metformin increases life expectancy via the insulin-signalling pathway, we measured the mRNA expression of Insulin/IGF-1 signalling pathway components that are required for the insulin-signalling pathway’s longevity effects. Onken and Driscol examined the effect of metformin on lifespan in daf-16 mutants. They found that daf-16 mutants grown on 50 mM metformin exhibited lifespan extension approximately equal to wild-type data and suggested that metformin may extend lifespan by a DAF-16/FOXO-independent mechanism (38). In our current study, in the presence of 100 mM metformin, *daf-16a*, *daf-16d/f* mRNA expression was significantly increased in N2 worms compared to the control group, while no significant change in daf-2 mRNA expression was observed. On the other hand, a statistically significant increase in mRNA expression of all Insulin/IGF-1 signalling components was observed in lin-35 worms compared to the control. Longevity in *C. elegans* is associated with increased expression of *daf16* and decreased expression of *daf-2*. We thus conclude that metformin may enhance lifespan via a DAF-16/FOXO-independent mechanism, which is consistent with the notion that metformin confers health-span advantages via a mechanism other than the insulin-like signalling pathway.

## 5 Conclusion

The present observations show that the negative effect of high glucose on the egg production of lin-35 worms was greater than that of N2 worms. At the same time, high glucose shortened the egg-laying time of individuals of both strains. In addition, high glucose shortened the lifespan and increased body length and width of individuals of both strains. Metformin treatment alone extended the lifespan of N2 and lin-35 worms by reducing fertilisation efficiency. The lifespan of N2 worms was further increased at lower concentrations (1 and 10 mM), while the lifespan of lin-35 worms was observed at higher metformin concentrations (10, 25 and 50 mM) compared to N2 worms. Moreover, when metformin was administered alone, the lifespan of lin-35 worms was extended to a greater extent than that of N2 worms. However, when metformin was administered in the presence of high glucose, the lifespan of lin-35 worms was clearly longer compared to N2 worms. In conditions where high glucose and metformin were administered together, the rate of increase in lifespan was greater in lin-35 worms compared to N2 worms in conditions where metformin alone was administered. Therefore, in the presence of high glucose and metformin, the lifespan of cancer mutant lin-35 worms was longer than N2 worms. This seems consistent with the data that metformin use slows down aging and extends life in patients with diabetes and/or aging-related diseases.

We conclude that in lin35 worms, glucose and metformin can extend life expectancy through a DAF-16/FOXO-independent mechanism. However, expression of IIS signalling pathway components reflects only mRNA levels and not actual protein levels. More research is needed on this topic, as mRNA levels do not necessarily reflect protein levels. It would also be useful to examine the protein expression levels of IIS components that play an active role in longevity and fertility processes. In addition, although it is known that the IIS signalling pathways are closely related to lifespan, the current study has shown for the first time how the combination of high glucose and metformin affects the lifespan of worms related with cancer. Moreover, the current study will be key in the literature to investigate the mechanistic pathway of high glucose and/or metformin treatment. Therefore, it will encourage more mechanism-oriented studies to confirm especially the qPCR data in our current study. Therefore, in the future, elucidating these mechanisms through molecular and biochemical experiments, especially in lin-35 worms with the cancer-related gene, will be useful in elucidating the *C. elegans* diabetes-cancer model.

## Data Availability

The raw data supporting the conclusions of this article will be made available by the authors, without undue reservation.
